# From Ancient Techniques to Modern Solutions: In Situ Synthesis of C‐S‐H for Sandstone Conservation

**DOI:** 10.1002/advs.202503333

**Published:** 2025-05-09

**Authors:** Mengjun Jia, Huimin Yan, Qingqing Xu, Celestino Grifa, Gang Zhao, Siwei Jiang, Jinhua Wang, Zhenhua Wei, Han Liu, Xiao Ma

**Affiliations:** ^1^ Department of History of Science and Scientific Archaeology University of Science and Technology of China Hefei 230026 China; ^2^ Institute for the Conservation of Cultural Heritage School of Cultural Heritage and Information Management Key Laboratory of Silicate Cultural Relics Conservation Ministry of Education Shanghai University Shanghai 200444 China; ^3^ Shanxi Museum Taiyuan 030024 China; ^4^ Department of Ocean Science and Engineering Southern University of Science and Technology Shenzhen 518055 China; ^5^ Dipartimento di Scienze e Tecnologie Università degli Studi del Sannio via De Sanctis snc Benevento 82100 Italy; ^6^ Academy of Dazu Rock Carvings Chongqing 402360 China; ^7^ Department of Cultural Heritage and Museology Fudan University Shanghai 200433 China; ^8^ SOlids in FormaTics AI‐Laboratory (SOFT‐AI‐Lab) College of Polymer Science and Engineering Sichuan University Chengdu 610065 China

**Keywords:** anti‐seepage, calcium‐silicate‐hydrate, grouting materials, mechanical properties, stone conservation

## Abstract

Hydraulic calcium‐silicate‐hydrate (C‐S‐H), a key binding agent in both ancient mortars and modern cement, holds significant promise for heritage conservation. Drawing inspiration from ancient Roman techniques, this study investigates the development of C‐S‐H‐based grouting materials to address water‐induced erosive damage in the Beishan Grottoes of the Dazu Rock Carvings. The interfacial interactions between C‐S‐H mortar and sandstone are analyzed using molecular dynamics simulations, revealing the crucial role of hydrogen bonding at the interface of C‐S‐H and mineral phases for adhesion. In the in situ synthesis of C‐S‐H with a reticulated structure from Ca(OH)_2_/silica fume mixtures under ambient conditions, this work systematically investigates the impact of varying calcium‐to‐silica (C/S) ratio and water‐to‐binder ratios on the mechanical properties and pore structure of C‐S‐H‐based mortars. The optimal mechanical and physical properties are achieved with a C/S ratio of 0.8, water/binder ratio of 2.0, binder/aggregate ratio of 1:3, and 4 wt% polycarboxylate superplasticizer. Laboratory‐scale experiments confirm its excellent compatibility with sandstone, offering a potential effective grouting solution for the Beishan Grottoes and emphasizing the importance of material compatibility in heritage conservation. This integrated approach encompassing materials design, synthesis, characterization, and interfacial analysis, presents a robust framework for developing tailored binding agents for various applications.

## Graphical abstract

Herein, this work presents a paleo‐inspired approach to synthesizing and optimizing C‐S‐H‐based grouting mortars for conserving the sandstone of Beishan Grottoes, Dazu Rock Carvings, China. By achieving an optimal recipe compatible with natural sandstone, this work demonstrates the pivotal role of hydrogen bonding in the adhesion between C‐S‐H mortars and minerals through integrated multi‐scale experimental analyses and molecular dynamics simulations.

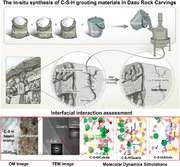

## 1 Introduction

Imagine standing beneath the magnificent dome of the Pantheon, a testament to the ingenuity of ancient engineers who harnessed the power of nature to create a structure that has withstood the test of time for nearly two millennia. At the heart of this enduring legacy lie hydraulic cementitious phases, specifically Calcium‐Silicate‐Hydrates (C‐S‐H) and Calcium‐Aluminum‐Silicate‐Hydrates (C‐A‐S‐H), formed from the pozzolanic reaction of lime, water, and reactive silicates or aluminosilicates. These materials have been foundational to construction throughout human history.^[^ ^1^ ^]^ Roman concrete and mortars stand out as prime examples of durable, resilient, and sustainable inorganic materials, boasting lifespans that exceed 2000 years.^[^ ^2^ ^]^ The Tomb of Caecilia Metella (30‐10 BCE), built with Roman concrete (C‐A‐S‐H binder) and dimension stone, highlights early Roman advancements in material technology and structural design.^[^ ^3^ ^]^ The Pantheon Dome (127 CE) remains the world's largest unreinforced concrete dome,^[^ ^4^ ^]^ while Pompeii showcases Roman mortars using “natural pozzolana” or “*cocciopesto*” aggregates.^[^ ^5^ ^]^ Remarkably, hydraulic materials (C‐S‐H) have been found at China's Dadiwan Site, dating back over 5000 years. This ancient mixture of lime, water, and burned calcareous nodules, known as “ginger nut” (料礓石), is considered the world's earliest “concrete.”^[^ ^6^ ^]^

Inspired by the durability of C‐S‐H in historical structures, we explored its potential as a grouting material for stone heritage.^[^ ^7^ ^]^ C‐S‐H is a poorly crystalline material characterized by a varied range of chemical compositions, consisting of calcium‐oxygen layers surrounded by silica chains and water interlayers.^[^ ^8^ ^]^ Its unique nanostructure contributes to exceptional mechanical and cohesive strength,^[^ ^9^ ^]^ establishing its crucial role in building construction throughout history. Additionally, its rapid‐hardening properties under aqueous or high‐humidity conditions make it ideal for water conservancy projects.^[^ ^10^ ^]^

Given its significant properties, C‐S‐H is also relevant in the conservation of historical structures, such as the grottoes. Grottoes are supported by geological formations, where various fractures developed in the rock provide pathways for groundwater seepage. Some of these water‐permeating fractures emerge within the sculpted areas of the caves, causing erosive damage to the stone carvings and sculptures,^[^ ^11^ ^]^ thereby posing a serious threat to their safety. To effectively mitigate the erosive damage caused by seepage, one of the effective measures is to use grouting technology on the threatening fractures. This technology involves drilling holes or using injection pipes to infuse anti‐seepage materials into the fissures, blocking the seepage pathways and eliminating the erosion damage induced by the seepage on the stone sculptures (Figure S1, Movie S1, Supporting Information).^[^ ^12^ ^]^

The success of grouting treatment relies significantly on the selection of appropriate grouting materials, which can be broadly categorized into organic and inorganic types. While organic materials like silicone,^[^ ^13^ ^]^ epoxy^[^ ^14^ ^]^ and acrylic acid resins^[^ ^15^ ^]^ offer good permeability and adhesion, their poor compatibility with hydrophilic stone matrices and limited durability restrict their use.^[^ ^11^, ^12^ ^]^ Inorganic materials like cement and natural hydraulic lime (NHLs) exhibit better compatibility and anti‐weathering properties^[^ ^11^, ^16^ ^]^ but face challenges such as high soluble salt content and insufficient strength development in enclosed environments.^[^ ^17^ ^]^

In light of these challenges, we drew inspiration from the primary hydration products of calcium‐silicate‐hydrate (C‐S‐H) found in historical buildings, modern cement and hydraulic β‐Ca_2_SiO_4_,^[^ ^18^ ^]^ which are renowned for their exceptional mechanical strength in high humidity conditions.^[^ ^19^ ^]^ The potential advantages of employing C‐S‐H as a binder for grouting material in stone heritage are significant in the following aspects: (a) C‐S‐H is rich in both CaO and SiO_2_ components, facilitating chemical bonding with silicates and carbonates within stone matrices; (b) C‐S‐H can form through hydraulic reactions, allowing it to harden in moist environments; (c) synthetic C‐S‐H, due to its high purity, minimizes soluble salts, which is advantageous for stone heritage conservation; and (d) the persistence of C‐S‐H in archaeological sites over millennia highlights its remarkable durability.^[^ ^5^ ^]^

Molecular dynamics simulations were used to analyze the chemical bond interactions between C‐S‐H and the predominant mineral constituents of sandstone, providing a theoretical basis for material selection and explaining the enhanced adhesive strength of C‐S‐H‐based mortars. Afterward, we embarked on synthesizing high‐purity C‐S‐H within a laboratory environment. Common synthesis methods for C‐S‐H encompass the hydrothermal method, utilizing pure CaO/Ca(OH)_2_ and silica, the liquid‐phase reaction method employing soluble calcium salt (e.g. calcium nitrate) and silicate (e.g. Na_2_SiO_3_·9H_2_O),^[^ ^20^ ^]^ hydration of calcium silicates (C_3_S or C_2_S),^[^ ^21^ ^]^ and the precipitation method involving soluble CaCl_2_ and Si(OH)_4_ titration with NaOH solution.^[^ ^22^ ^]^ However, soluble salts (e.g., Na⁺, Cl⁻, NO₃⁻) are unsuitable for conservation due to potential salt damage,^[^ ^23^ ^]^ and calcium silicate hydration requires high energy consumption.^[^ ^24^ ^]^ Recognizing the critical need for in situ synthesis of C‐S‐H at room temperature, this study draws inspiration from ancient Roman pozzolanic techniques. Pozzolana‐lime cements, first used in regions like Pompeii and Puteoli, were made by mixing pozzolana (volcanic ash) with lime and water.^[^ ^25^ ^]^ These cements were employed in large‐scale maritime and structural projects due to their exceptional bonding properties. The earliest evidence of pozzolana‐lime cement dates to the third century BC in Pompeii, while the harbor works of Puteoli remain remarkably well‐preserved despite harsh marine conditions.^[^ ^25a^ ^]^ In this study, we replicated this ancient method by combining lime (Ca(OH)₂) and fumed silica to prepare hydraulic mortars.

The chemical composition and resultant micro‐ and nanostructure characteristics of synthesized C‐S‐H are significantly influenced by the Ca/Si molar ratio and water content.^[^ ^8^, ^26^ ^]^ In this study, C‐S‐H binders were synthesized by mixing Ca(OH)_2_ and active silica fume at ambient temperature. The research focused on examining the effects of the Ca/Si ratio and the water‐to‐binder ratio on the yield of C‐S‐H gel, as well as the factors determining the mechanical strength of C‐S‐H‐based mortars. With the aim of formulating a suitable C‐S‐H‐based mortar recipe that balances good workability with sufficient mechanical strength for the conservation of Beishan sandstone at the Dazu Rock Carvings, we assessed the compatibility of the grouting mortar with natural sandstones. In conclusion, we demonstrate that synthetic C‐S‐H represents a suitable binder for grouting materials in the conservation of stone heritage, offering a promising solution to the conservation challenges faced by significant historical sites.

## 2 Results and Discussion

### 2.1 Interface and Chemical Bonding between the C‐S‐H Material and Sandstone

Effective chemical bonding between grouting materials and stone heritage is crucial to the performance of stone conservation efforts. While it is well‐established that C‐S‐H chemically interacts with aggregates in concrete structures, leading to enhanced macroscopic performance, the interactions between hydraulic C‐S‐H materials and natural sandstone—a complex mixture of minerals—remain less understood. It is essential to first understand the atomic‐scale chemical interactions between C‐S‐H and the underlying stone matrix.

Therefore, in this study, we employed molecular dynamics simulations to investigate the chemical interaction bonds at the interface between C‐S‐H and principal mineral phases (here, we used the sandstone samples from the Beishan Grottoes as example, which are composed primarily of quartz, calcite, and albite, as determined through composition analysis, see Figure S2, Supporting Information). A detailed analysis of the chemical interactions between the C‐S‐H phases and each of the three predominant sandstone components—quartz, calcite, and albite—was conducted individually. The hardened state of grouting material, characterized by the hydrated calcium silicate hydrate (C‐S‐H) phase as the primary hydration product, represents the stable binding phase in fully hydrated cement systems with well‐defined structural properties and interfacial bonding characteristics.

**Figure 1:**
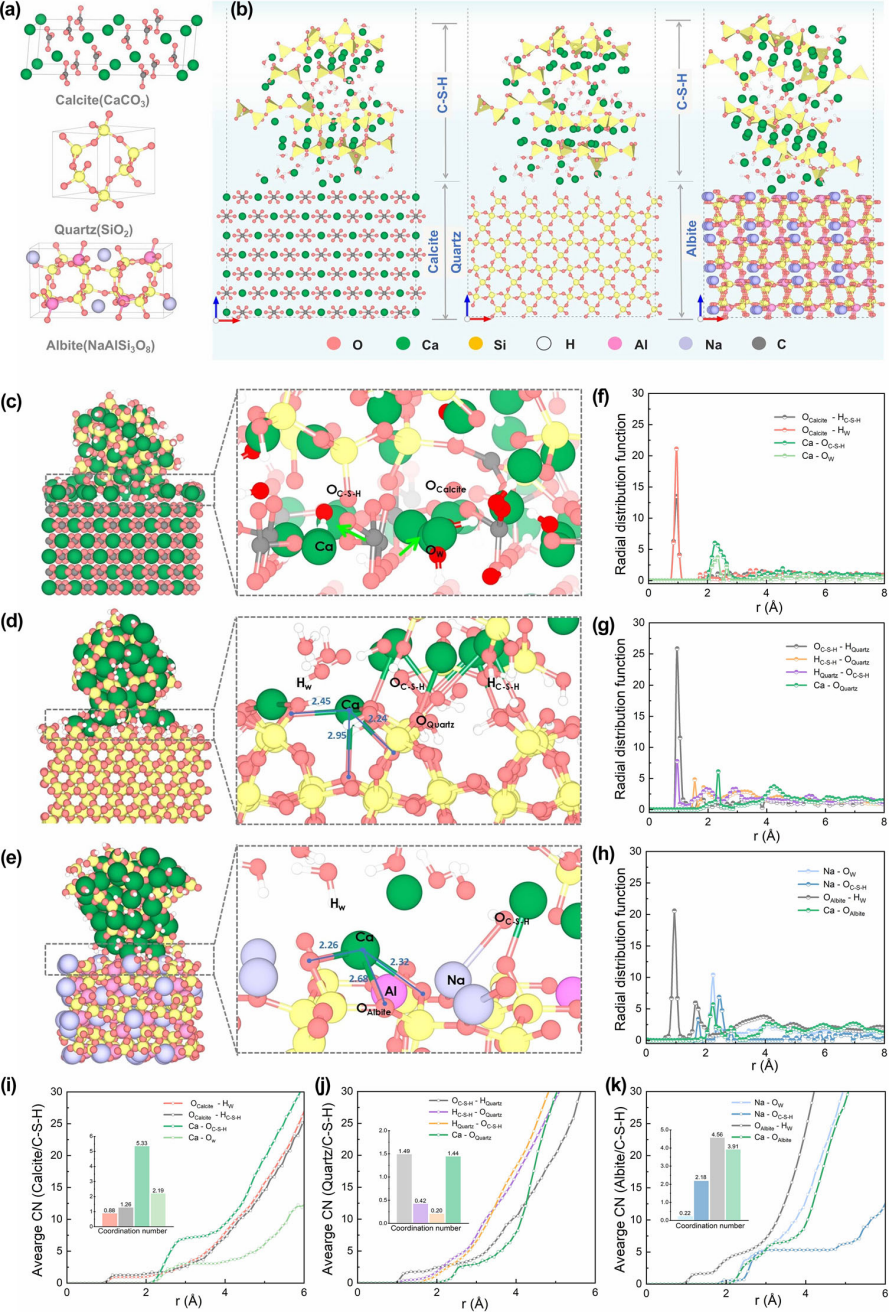
a) Base models of calcite, quartz and albite. b) Composite molecular dynamics models of calcite/C‐S‐H, quartz/C‐S‐H and albite/C‐S‐H. c–e) Details of the equilibrium states for calcite/C‐S‐H, quartz/C‐S‐H and albite/C‐S‐H models. f–h) RDF profiles of the calcite/C‐S‐H, quartz/C‐S‐H and albite/C‐S‐H interfaces. i–k) Average coordination number for the calcite/C‐S‐H, quartz/C‐S‐H and albite/C‐S‐H models.

To ensure consistency with experimental parameters, models for calcite, alpha‐quartz and albite were developed, as illustrated in **Figure** **1**a. The composite molecular dynamics models of calcite/C‐S‐H, quartz/C‐S‐H, and albite/C‐S‐H, shown in Figure 1b, resulted in composite dimensions of 24.28 Å × 29.93 Å × 64.77 Å, 29.47 Å × 21.62 Å × 63.55 Å, and 23.15 Å × 25.12 Å × 58.68 Å, respectively. The interfacial stability model and details of the equilibrium state are presented in Figure 1c–e, which include the atoms in the C‐S‐H silica chain (O_Si_, H_Si_), and the water molecules (H_W_ and O_W_).

Figure 1c illustrates the bonding interactions at the interface between calcite and C‐S‐H interface. It shows that both Ca atoms from C‐S‐H (Ca_C‐S‐H_) and calcite (Ca_calcite_) exhibit distinct degrees of displacement, indicated by an arrow representing their movement directions. Specifically, Ca_C‐S‐H_ moves downward, occupying the position previously held by Ca_calcite_. This shift facilitates the bonding of Ca_calcite_ with O_W_ and O_Si_ in C‐S‐H. The primary bonding mechanism at the interface is the interaction between Ca_calcite_ and O_Si_, as well as between Ca_calcite_ and O_W_. In the interfacial bonding details illustrated in Figure 1d, C‐S‐H is shown to form strong bonds with the quartz matrix through chemical interactions. Key atoms, specifically O_quartz_ and H_quartz_ on the quartz surface, create attractive interactions with Ca, H, and O in C‐S‐H. As the simulation progressed, calcium atoms migrated to energetically favorable positions, forming Ca‐O bonds with surface oxygen atoms of quartz, with characteristic bond lengths between 2.24 and 2.95 Å. Figure 1e reveals the presence of bonds between the oxygen atoms in C‐S‐H and sodium (Na) ions at the interface. However, calcium and oxygen demonstrate stronger attractive interactions, forming Ca‐O bonds with lengths ranging from 2.26 to 2.68 Å. Additionally, due to adsorption, the bond angles of water molecules adopt an inverted ‘V’ shape, with H_W_ oriented toward the surface oxygen. The presence of Ca^2^⁺ and Na⁺ cations significantly modulates the hydrogen bonding network at the interface. These cations exert distinct influences through their charge‐driven coordination behavior and competitive hydration effects. Specifically, Ca^2^⁺ ions enhance interfacial cohesion by stabilizing oriented hydrogen bonds through their strong electrostatic interactions. In contrast, Na⁺ ions preferentially promote water‐mediated hydrogen bonding due to their weaker direct coordination but stronger hydration shell formation. These differential mechanisms lead to fundamentally distinct interfacial mechanical properties and chemical stability, providing crucial atomistic‐level insights for the rational design of grout materials with tailored interfacial characteristics.

The Radial Distribution Function (RDF) of atoms at the interface was statistically calculated to elucidate the interfacial structures between C‐S‐H and sandstones. Figure 1f–h displays the RDF curves for calcite/C‐S‐H, quartz/C‐S‐H and albite/C‐S‐H, respectively. Figure 1f shows the first peak of RDF between hydrogen atoms on the C‐S‐H surface (H_Si_, H_W_) and oxygen atoms (O_calcite_) on the calcite surface at 0.95 Å. Similarly, the RDF between H_W_ and oxygen atoms in albite (O_albite_) also exhibits a prominent peak at 0.95 Å (Figure 1h). Furthermore, Figure 1g reveals three distinct peaks for H_quartz_‐O_Si_, O_quartz_‐H_W_ and H_quartz_‐O_W_ at 0.95 Å, 0.95 Å, and 1.55 Å, respectively, all within the hydrogen bonding distance threshold.^[^ ^27^ ^]^ This indicates that the hydrogen bonding network significantly influences the bonding at the C‐S‐H and sandstone interface. Additionally, the characteristic peaks of the RDF curves for Ca_C‐S‐H_‐O_albite_ and Ca_C‐S‐H_‐O_quartz_ were identified at 2.25 and 2.35 Å, respectively, consistent with typical metal‐oxygen bond distance. This suggests that the protected sandstone provides numerous oxygen sites for calcium atoms in C‐S‐H. At the C‐S‐H and calcite interface, the peak for Ca_calcite_‐O_Si_ is more pronounced than that for Ca_calcite_‐O_W_ indicating that water molecules are distributed near calcite at the interface, although the interaction with Ca_calcite_‐O_W_ is relatively weak. In the RDF curve at the albite and C‐S‐H interface, the binding of Na_albite_‐O_Si_ and Na_albite_‐O_w_ is observed at 1.75 Å and 2.25 Å, reflecting attractive interactions that are also evident in the sub‐interface diagram.

The first crossing of the RDF curve and the coordination number (CN) curve indicate that the coordinating atoms are counted in the coordination number within this distance. To quantify bonding, we compared the average coordination number at the interface, as shown in Figure 1i–k. In the calcite/C‐S‐H system, the coordination numbers for Ca_calcite_‐O_Si _and Ca_calcite_‐O_W_ under stable conditions were determined to be 5.33 and 2.19, respectively, based on the stable plateau of the coordination number curve. These results suggest that calcium ions primarily interact with oxygen atoms on the silicate chains. Additionally, the coordination of hydrogen on the silicate chains with oxygen from calcite indicates an interface merging, where C‐S‐H and calcite are closely linked, with water molecules providing binding sites. In Figure 1j, both O_Si_‐H_quartz_ and Ca_C‐S‐H_‐O_quartz_ exhibit relatively high coordination numbers, suggesting that interactions involve hydrogen bonding and variations in calcium‐oxygen coordination as calcium ions migrate within the system. At the albite/C‐S‐H interface, the coordination number of Na_albite_‐O_W_ stabilizes at 0.22, indicating limited coordination of Na with oxygen. The highest coordination is observed for O_albite_‐H_W_, suggesting that O_albite_ primarily interacts with surface water, leading to stable adsorption at the interface. This atomic interaction contributes to greater structural compactness within the system.

Molecular dynamics simulations analysis confirms that the formation of hydrogen bonds between oxygen and hydrogen atoms in C‐S‐H or mineral phases is the primary contributor to the interaction between the binders in the grouting materials and stone matrices. Notably, the identified Ca_C‐S‐H_‐O_quartz_ and Ca_C‐S‐H_‐O_albite_ bonding configuration provide new insights into the interfacial interactions between C‐S‐H and silicate minerals in stone conservatin applications. This observation highlights the potential role of active calcium ions in facilitating the binding between C‐S‐H and various silicate mineral phases, which is crucial for understanding the compatibility of grouting materials used in the conservation of stone heritage. The presence of these ions may enhance the adhesive properties of the binder, ensuring a robust and enduring interaction with the mineral components of the stone, which is essential for the long‐term stability and integrity of the grouting materials.

### 2.2 The Synthesis of Calcium‐Silicate‐Hydrate

**Figure 2:**
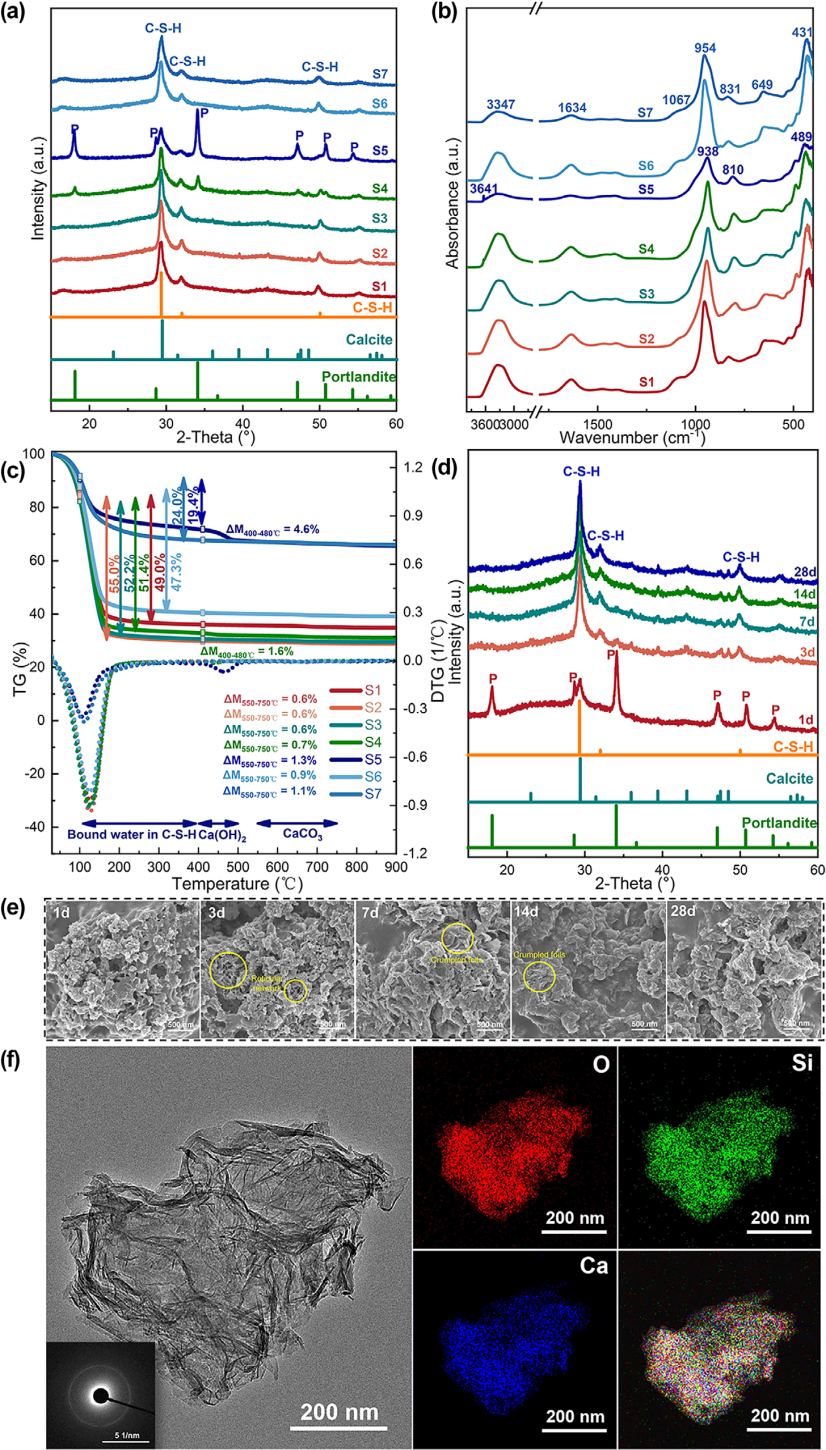
Compositional and morphological analysis of ambient‐synthesized C‐S‐H. a) XRD, b) FTIR, and c) TG‐DTG analysis of C‐S‐H (S1–S7) samples synthesized at ambient conditions; d) XRD and e) the SEM images of the C‐S‐H sample with an initial C/S ratio of 1.0 and water/binder ratio of 2.0 after curing for 1 day, 3 days, 7 days, 14 days and 28 days, respectively; f) TEM image and elements mapping of synthesized C‐S‐H.

The mechanism for C‐S‐H gel formation through the pozzolanic reaction between Ca(OH)_2_ and amorphous silica involves several steps, including: 1) Chemisorption of Ca(OH)_2_ by the surface silanol groups of silica; 2) Silica reacting with water to generate a saturated solution of monosilicic acid; 3) The reaction of monosilicic acid or its ions with Ca(OH)_2_ to produce C‐S‐H in solution; 4) Nucleation, growth, flocculation and precipitation of C‐S‐H crystals, following Greenberg's model. Among these steps, the dissoltuion of silica (the second step) is believed to regulate the overall kinetics, highlighting the importance of an active silica source in C‐S‐H synthesis.^[^ ^28^ ^]^ In our preliminary experiments, we compared and screened silica fume (see Section *Materials*) due to its higher reactivity with Ca(OH)_2_ and cost‐effectiveness, making it suitable for large‐scale application. Numerous studies have demonstrated that both the Ca(OH)₂/silica molar ratio (C/S ratio) and the water‐to‐binder ratio significantly influence the microstructure and morphology of calcium silicate hydrate (C‐S‐H). These factors, in turn, profoundly affect the macroscopic properties of the material.^[^ ^8^, ^26^ ^]^ Therefore, this section investigated three factors of C/S molar ratio, water/binder ratio (water/binder ratio), and reaction time to optimize the conditions for the synthesis of Calcium‐Silicate‐Hydrate (C‐S‐H). To determine the suitable C/S molar ratio, mixtures labeled as S1 to S5, consisting of Ca(OH)_2_, silica fume (a commercial amorphous silica source was selected, see Figure S3, Supporting Information) and water, were prepared with C/S ratios ranging from 0.8 to 2.0 while maintaining a fixed water/binder ratio of 4.0. These mixtures were analyzed by TEM, XRD, FTIR and TGA‐DSC after curing for 7 days. **Figure** **2**f indicates that the C‐S‐H synthesized at ambient conditions is a poor‐ordered phase composed of three main elements: Ca, O and Si. The elemental mapping shows a complete overlap between these elements, corroborating the findings of the XRD analysis and FTIR analysis (Figure 2a,b), confirming the presence of C‐S‐H phase. XRD results showed the peaks of C‐S‐H (with peaks at 2θ = 29.5°, 32.3° and 50.0°) in all samples. The samples S4 and S5, with a C/S ratio of 1.5–2.0, also showed peaks corresponding to excess Ca(OH)_2_ (at 2θ = 18.1°, 28.7°, and 34.1°). Although the peak at 2θ = 29.5° overlaps for C‐S‐H and calcite phase of CaCO_3_,^[^ ^29^ ^]^ the absence of other peaks corresponding to calcite suggested the prevalence of C‐S‐H over calcite. The FTIR analysis results were consistent with XRD analysis. Peaks observed at 3347 and 1634 cm^−1^ correspond to both the bound water in C‐S‐H and the surface‐absorbed water. Several peaks between 1200–800 cm^−1^ are associated with the asymmetric and symmetric stretching vibrations of Si‐O bonds in C‐S‐H structures. The bands at 649 cm^−1^ and in the range of 400–500 cm^−1^ correspond to the Si‐O‐Si bending vibrations and the deformation of SiO_4_ tetrahedra, respectively.^[^ ^30^ ^]^ Additionally, small peaks at 3641 cm^−1^ illustrate the presence of excess Ca(OH)_2_ in samples S4 and S5.

Figure 2c and **Table** **1** show the TG‐DTG analysis of the samples that have been cured for 7 days. The water loss between 105 °C and the onset of Ca(OH)_2_ dehydration is considered as the water loss in C‐S‐H.^[^ ^10^, ^31^ ^]^ The mass of bound water in C‐S‐H increases as the initial C/S ratio decreases for samples S2–S5 (55.0–19.4 wt. %). The fine water loss between 400–480 °C indicated excess Ca(OH)_2_ in the samples S4 and S5, which is consistent with XRD and FTIR results (Figure 2a,b). A slight mass loss between 550–750 °C (0.6–1.3 wt. %) was associated with CO_2_ release from calcium carbonate, indicating a relatively low amount of CaCO_3_ (1.4–3.0 wt. %) formed during the mixing and curing process. In conclusion, C‐S‐H primarily formed in the wet mixtures of Ca(OH)_2_ and amorphous silica, with a higher amount of yield obtained at lower C/S ratios.

**Table 1:** TGA analysis of ambient‐synthesized C‐S‐H samples.

No.	Initial C/S	water/binder ratio	TGA [wt%]		
			Dehydration of C‐S‐H	Mass of Ca(OH)_2_	Mass of CaCO_3_
			105–400 °C	400–480 °C	550–750°C
**S1**	0.8	4.0	49.0	0.0	1.4
**S2**	1.0	4.0	55.0	0.0	1.4
**S3**	1.2	4.0	52.2	0.0	1.4
**S4**	1.5	4.0	51.4	6.6	1.6
**S5**	2.0	4.0	19.4	18.9	3.0
**S6**	0.8	2.0	47.3	0.0	2.0
**S7**	0.8	6.0	24.0	0.0	2.5

Given the significant impact of the water/binder ratio on both the synthesis of C‐S‐H and the consistency of C‐S‐H based grouting mortar, we investigated the samples S1, S6 and S7 with a C/S ratio of 0.8 and water/binder ratios ranging from 2 to 6. Figure 2c and Table 1 show that all three samples formed the C‐S‐H phase with a small amount of CaCO_3_ (1.4–2.5%). Given that a lower water/binder ratio is crucial for the consistency of grouting mortars and enhances mechanical strength,^[^ ^32^ ^]^ while also helping to avoid water‐related issues such as water seepage or salt weathering, a water/binder ratio of 2.0 was selected for the preparation of C‐S‐H based mortars in subsequent research.

Additional investigations into the formation of C‐S‐H phases in the Ca(OH)_2_/Silica fume mixture, featuring a C/S ratio of 1.0 and a water/binder ratio of 2.0, over curing durations of 1, 3, 7, 14, and 28 days under ambient conditions, revealed that the pozzolanic reaction between Ca(OH)_2_ and silica fume occurs rapidly. The exclusive presence of C‐S‐H phase was observed in the mixtures after 3 days (Figure 2d). The absence of discernible Ca(OH)_2_ peaks in the XRD, along with the emergence of reticular networked phases after 3 days and the presence of crumpled foils or a denser C‐S‐H structure during 7–28 days period (Figure 2d,e), indicate the swift production of C‐S‐H in the ambient‐cured mixtures. This rapid formation could prove advantageous for C‐S‐H‐based mortars, particularly when utilized as grouting materials.

### 2.3 Composition and Mechanical Properties of C‐S‐H based Mortars and Their Compatibility with Sandstones from Beishan Grottoes of Dazu Rock Carvings

**Figure 3:**
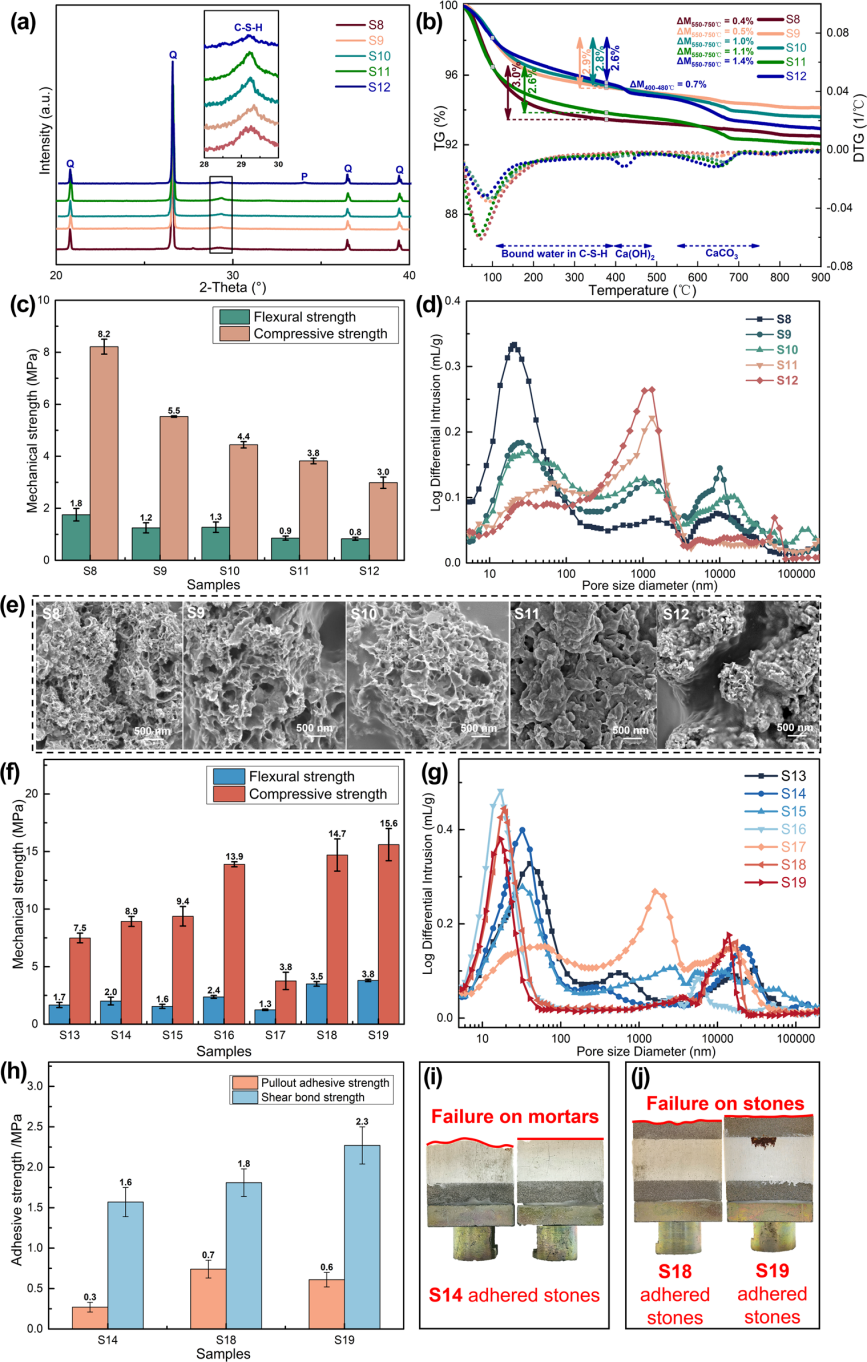
Compositional, morphological analysis and macro‐properties of C‐S‐H based mortars. a) XRD analysis, b) TG‐DTG analysis and c) flexural/compressive strength, d) pore size distribution and e) SEM images of C‐S‐H based mortars (samples S8–S12) cured for 28 days; f) Flexural/compressive strength and g) pore size distribution of C‐S‐H based mortars (samples S13–S19) cured for 28 days; h) The adhesive strength of samples S14, S18 and S19 samples with Dazu Rock Carvings sandstones and i, j) the failure patterns of the adhered sandstones during pull out strength test.

In order to find the optimum parameters for the preparation of grouting mortars, we investigated the composition and mechanical properties of the C‐S‐H mortars prepared by the mixture of Ca(OH)_2_, silica fume and quartz aggregates. To study the composition of C‐S‐H based mortars, we conducted the XRD and TG‐DTG analyses for C‐S‐H based mortars (S8–S12) with C/S ratio of 0.8–2.0, a water/binder ratio of 2.0 and a binder/aggregate ratio of 1:3. The peaks of 2θ = 29.5° in XRD patterns indicate the presence of C‐S‐H phases in all the cured samples (**Figure** **3**a). The amount of structural H_2_O in C‐S‐H (3.0–2.6 wt. %, Figure 3b) between 105–400 °C decreases with an increase in C/S ratio (from S8 to S12), illustrating the yield of C‐S‐H increases with the decreasing of C/S ratio, consistent with previous research.

The mechanical strength of the ambient‐synthetic C‐S‐H‐based mortars was assessed through flexural and compressive strength tests in conjunction with pore size distribution (PSD) characterization. Subsequently, this section fine‐tuned and assessed the workability and compatibility with natural sandstones of the C‐S‐H‐based mortars.

The flexural strength and compressive strength of C‐S‐H‐based mortars were measured and compared. Figure 3c and **Table** **2** illustrate both flexural strength (decreasing from 1.8 to 0.8 MPa) and compressive strength (decreasing from 8.2 to 3.0 MPa) with an increasing C/S ratio ranging from 0.8–2.0 (samples S8–S12). This trend aligns with the findings in composition analysis (Figure 2a,b and Table 1), which indicates that the yield of C‐S‐H increases as the C/S ratio decreases. It can be inferred that the optimal mechanical strength is associated with a high‐yield C‐S‐H binder in the mortars (C/S = 0.8). Additionally, the micro‐pore structure constitutes another crucial factor influencing mechanical strength. Pore size distribution curves (Figure 3d) reveal that all C‐S‐H based mortars (samples S8–S12) exhibit meso‐pores, with a predominant size ≈20–30 nm, and macro‐pores, with a predominant size ≈1–2 µm or larger than 10 µm. As the C/S increases, the intensity of meso‐pores gradually decreases, while the presence of macro‐pores between 1–2 µm increases significantly, particularly when C/S > 1.5 (samples S11 and S12). These findings suggest that the high mechanical strength observed in the formulations with a low C/S ratio may be attributed to a matrix characterized by a higher presence of meso‐pores and a lower abundance of macro‐pores, resulting from the higher yield of C‐S‐H binders with aggregates. Despite C‐S‐H based mortars with a C/S of 2.0 displaying the lowest porosity (47%), the lower yield of C‐S‐H binders (Figure 3b and Table S1, Supporting Information) and the presence of more macro‐pores (0.2–3 µm, Figure 3d) in the matrix contribute to its inferior mechanical strength (Figure 3c).

**Table 2:** Mechanical, physical properties and the workability of C‐S‐H based mortars.

No.	Initial C/S	Amount of PCE [wt%]	water/ binder ratio	binder/ aggregate ratio	Fluidity [mm]	Setting time [min]	Compressive Strength [MPa]	Flexural Strength [MPa]	Porosity [%]
**S8**	0.8	0	2.0	1:3	‐	371	8.2 ± 0.3	1.8 ± 0.2	50
**S9**	1.0					‐	5.5 ± 0.1	1.2 ± 0.2	50
**S10**	1.2						4.4 ± 0.1	1.3 ± 0.2	52
**S11**	1.5						3.8 ± 0.1	0.9 ± 0.1	49
**S12**	2.0						3.0 ± 0.2	0.8 ± 0.1	47
**S13**	0.8	3			145 ± 2	420	7.5 ± 0.4	1.7 ± 0.2	52
**S14**		4			259 ± 3	491	8.9 ± 0.4	2.0 ± 0.3	51
**S15**		5			264 ± 3	550	9.4 ± 0.8	1.6 ± 0.2	52
**S16**		4	1.5		‐	227	13.9 ± 0.2	2.4 ± 0.1	45
**S17**		4	2.5		295 ± 2	883	3.8 ± 0.8	1.3 ± 0.1	55
**S18**		5	1.5		201 ± 1	184	14.7 ± 1.4	3.5 ± 0.2	42
**S19**		7	1.4		213 ± 2	319	15.6 ± 1.4	3.8 ± 0.1	38

Certainly, the microstructural features of C‐S‐H phases in the cured mortars play a crucial role in determining the final pore structures. Figure 3e illustrates the characteristic reticular network structures observed when C/S is below 1.2 (samples S8–S10). However, as the C/S ratio increases, the network becomes less densely packed. Moreover, when the C/S ratio exceeds 1.5, the continuity of C‐S‐H phase is constrained by the by‐products of CaCO_3_ or un‐reacted Ca(OH)_2_ (Figure 3b and Table 1). This results in a less uniform matrix with numerous large macro‐pores or fissures (samples S11 and S12, Figure 3e). Consequently, the denser reticular network of C‐S‐H and the homogeneous structure in the cured matrix with a lower C/S ratio are likely to influence the mechanical properties positively. Given that the rocks used in the construction of grotto temples typically exhibit compressive strengths in the range of tens of MPa, a C/S ratio of 0.8 was selected. This ratio maximizes the yield of C‐S‐H products and promotes a denser pore structure, thereby optimizing the strength compatibility of the C‐S‐H‐based materials.

To improve the workability of C‐S‐H‐based mortars to meet the specifications for use as grouting mortars, adjustments were made to both the superplasticizer dosage and the water/binder ratio in this section. In general, insufficient water is detrimental to the synthesis of C‐S‐H binders, while excessive water negatively impacts on the macro‐properties of cured mortars^[^ ^33^ ^]^ and may potentially give rise to other issues, such as salt movement.^[^ ^34^ ^]^ Therefore, to achieve suitable workability, polycarboxylate superplasticizer (PCE) was introduced to reduce unnecessary water content while maintaining the essential water for C‐S‐H formation. This section compares the fluidity and setting time of C‐S‐H‐based mortars, along with the compressive/flexural strength and pore structures of the cured C‐S‐H‐based mortars (samples S13‐S19).

Table 2 demonstrates that C‐S‐H mortars with a water/binder ratio of 2 (samples S13–S15), containing more than 4 wt. % PCE exhibit excellent workability, with fluidity ranging from 259 to 264 mm. However, the setting time for these mortars is extended, increasing from 371 min (sample S8) to 420–550 min (samples S13‐S15) as the PCE content increases from 3 wt. % to 5 wt. %. Additionally, it is observed that the introduction of more water (samples S16, S14 and S17) significantly enhances mortar fluidity (259–295 mm) and delays the setting time (227–883 min). This highlights the effectiveness of both superplasticizers and water in modifying the workability of C‐S‐H‐based mortars.

In the context of C‐S‐H‐based mortars, the addition of PCE and water play distinct roles in influencing the final mechanical strength and pore structures. In comparison to the sample S8 with flexural/compressive strength of 1.8/8.2 MPa (Figure 3c and Table 2), there is only a marginal improvement in compressive/flexural strength when the PCE content exceeds 4 wt. % (sample S14 with flexural/compressive strength of 2.0/8.9 MPa). However, a decreasing trend in flexural strength is observed for C‐S‐H‐based mortar sample S15 (1.6 MPa) when PCE content reaches 5 wt. % (Figure 3f). The dispersion mechanism of polycarboxylate superplasticizer (PCE) is closely related to its chemical structure. The main chain of PCE, which contains numerous anionic groups, coordinates with Ca^2+^ ions on the surface of calcium hydroxide (Ca(OH)_2_) particles. Simultaneously, the flexible side chains of PCE form an adsorption layer of a certain thickness on the surface of these particles. When adjacent Ca(OH)_2_ particles come close to each other, the thickness of the overlapping adsorption layers increases. This overlap generates significant steric repulsion, which effectively disperses the particles and prevents their aggregation to improve the workability of the fresh mortars.^[^ ^35^ ^]^ In the current material system, the water‐reducing effect of PCE is primarily attributed to the coordination of carboxyl groups with Ca^2+^ on the surface of Ca(OH)_2_ particles. Simultaneously, the consistency of C‐S‐H‐based grouting mortars depends on the production of C‐S‐H as a medium to bind the sand aggregates together. In this scenario, there is a competition between PCE and silica fume, both of which are absorbed by the Ca(OH)_2_ particles. Consequently, excessive PCE may hinder the reaction between Ca(OH)_2_ and silica fume,^[^ ^36^ ^]^ resulting in a reduced yield of C‐S‐H binders (Figure S4a, Supporting Information) necessary for effective cohesion between the aggregates, as observed in sample S15. The addition of PCE has a minimal effect on the composition and microstructure of C‐S‐H, as evidenced by XRD and SEM analysis (Figure S4b,c). It only leads to a slight increase in porosity (samples S13‐S15, 51%–52%, Table 2) and has a minor impact on small‐sized pores (samples S13‐S15,<200 nm, Figure 3g) compared to the mortar sample S8 (Figure 3d).

Water appears to have a more pronounced effect on the macro‐properties of C‐S‐H‐based grouting mortars compared to PCE. First, with a water/binder ratio of 1.5–2.5 (samples S16, S14 and S17), both compressive and flexural strength of the mortar samples exhibit a noticeable decreasing trend with increasing water content (Figure 3f and Table 2). Second, the addition of water leads to a significant increase in porosity (45–55%) and a noticeable shift in mesopore size from 20 to 60 nm (samples S16, S14, and S17; Figure 3g). In sample S17, which has a higher water/binder ratio of 2.5, the appearance of macropores (1–3 µm) indicates reduced cohesion between the sand aggregates. This results in the lowest flexural and compressive strengths of 1.3 MPa and 3.8 MPa, respectively (Figure 3f and Table 2). From a microscale perspective, a higher water/binder ratio (e.g., S17) reduces the yield of C‐S‐H, as evidenced by XRD and TG‐DTG analysis. This leads to a less dense pore structure, as shown in Figure S5, Supporting Information. In contrast, a lower water/binder ratio promotes a higher yield of calcium silicate hydrate (C‐S‐H), resulting in a denser pore structure and enhanced macroscopic mechanical strength.

However, to assess the suitability of C‐S‐H‐based mortars as grouting materials for filling the fractures or cracks in sandstones of Beishan Grottoes of Dazu Rock Carving, additional evaluation on the composition, microstructure and properties of sandstones were conducted. Furthermore, pullout adhesive strength (PAS) and shear bond strength (SBS) tests with sandstone were performed (Figure S6, Supporting Information).

To figure out the appropriate mechanical strength of the grouting mortar compatible with the sandstone and to further balance the workability and mechanical strength for C‐S‐H‐based mortars, mixtures (samples S18‐S19) with water/binder ratios of 1.5–1.4 and PCE content of 5–7 wt. % were prepared. Table 2 indicates that with a higher amount of PCE, both samples S18 and S19 achieve a good fluidity higher than 200 mm. Meanwhile, the flexural/compressive strength of samples S18 (3.5/14.7 MPa) and S19 (3.8/15.6 MPa) show significant improvement (Figure 3f). Additionally, it is evident that the pore structure of S18 and S19 are more compacted, with a much lower porosity of 42–38% (Table 2) and more small‐sized pores (<60 nm, Figure 3g). Therefore, we have identified three recipes of C‐S‐H‐based mortars (samples S14, S18 and S19) with both good workability (Fluidity > 200 mm) and sufficient mechanical strength (Flexural/compressive strength > 2.0/8.9 MPa).

All three samples S14, S18 and S19 are selected for pullout adhesive strength (PAS) and shear bond strength (SBS) tests. From the results of PAS and SBS tests, it is observed that the pullout adhesive strength/shear bond strength of samples S18 (0.7/1.8 MPa) and S19 (0.6/2.3 MPa) surpass those of sample S14 (0.3/1.6 MPa, Figure 3h). However, a noteworthy observation is that the failure of sandstones adhered with samples S18 and S19 occurs within the sandstone matrix (Figure 3j), which contradicts the principles of cultural heritage conservation.^[^ ^37^ ^]^ Conversely, in the case of sandstones bonded with sample S14, failure predominantly occurs within the mortar bulk or at the interface between the sandstone and mortar, which is a more desirable outcome for grouting materials (Figure 3i).

The coefficient of permeability of the S14 was measured to be ≈8.8 × 10^−12^ cm s^−1^, indicating its excellent waterproofing properties and making it suitable for applications where water resistance is critical.

Based on the current results, one suitable formulation for use as grouting materials for water seepage of the sandstones of Beishan Grottoes of Dazu Rock Carvings is sample S14. This recipe provides appropriate mechanical strength and exhibits enhanced compatibility with the sandstones in the current study.

The interfaces between the samples S14 and the sandstone of Beishan Grottoes of Dazu Rock Carvings is shown in Figure S7, Supporting Information. Panels S7a‐c illustrate the strong macroscopic adhesion between the sandstone matrix and C‐S‐H‐based mortars. Elemental mapping reveals direct contact between C‐S‐H and the mineral phases, including quartz, calcite, and albite, which are the principal constituents of sandstone from Beishan of Dazu Rock Carvings (Figure S7d, Supporting Information). Further HR‐TEM analysis of the interface between C‐S‐H and quartz indicates atomic‐scale connectivity between the two phases (Figure S7e, Supporting Information), which is believed to facilitate the formation of new interfacial bonds, as analyzed in the molecular dynamics study above (Figure 1).

## 3 Conclusion

The synthesis of hydrated calcium silicate (C‐S‐H) with a reticular network has been achieved through the pozzolanic reaction in Ca(OH)_2_/silica fume mixtures under ambient conditions. Both the C/S ratio and water/binder ratio play crucial roles in determining the yield of synthetic C‐S‐H as well as the subsequent mechanical strength and pore structure of the C‐S‐H based mortars. As the C/S ratio decreases, there is a corresponding increase in the yield of C‐S‐H. Notably, mortars with a C/S ratio of 0.8 and a water/binder ratio of 2.0 exhibit the highest flexural/compressive strength of 1.8/8.2 MPa and denser pore structures with a high concentration of pores less than 200 nm in this study.

In the pursuit of a qualified C‐S‐H‐based grouting material with good workability and compatibility with natural stones, adjustments were made with the polycarboxylate superplasticizer (PCE) and water/binder ratio. Mortars with a C/S ratio of 0.8, water/binder ratio of 2.0, binder/aggregate ratio of 1:3 supplemented with 4 wt.% PCE, demonstrated a suitable fluidity (259 mm) and setting time (491 min), as well as appropriate flexural/compressive strength of 2.0/8.9 MPa. Additionally, these mortars exhibited undamaged pullout adhesive strength/shear bond strength of 0.3/1.6 MPa with the protected sandstones.

Molecular dynamics simulations analysis confirms that the formation of hydrogen bonds between oxygen and hydrogen atoms in C‐S‐H or mineral phases is the primary contributor to the interaction between the binders in the grouting materials and stone matrices. Notably, our simulations identify characteristic interfacial bonding configurations between Ca atoms in C‐S‐H and oxygen sites in both quartz (Ca‐O_quartz_) and albite (Ca‐O_albite_). This observation highlights the potential role of active calcium ions in facilitating the binding between C‐S‐H and various silicate mineral phases, which is crucial for understanding the compatibility of grouting materials used in the conservation of stone heritage. The presence of these ions may enhance the adhesive properties of the binder, ensuring a robust and enduring interaction with the mineral components of the stone, which is essential for the long‐term stability and integrity of the grouting materials.

This research demonstrates the potential application of grouting materials for sandstone conservation, highlighting the importance of compatibility between conservation materials and protected heritage matrix in material design. However, the specificity of stone material, in terms of mineralogical, microtextural, geochemical and physical‐mechanical features must also be carefully taken into account. To this aim, further research on studies of rocks in Beishan and Baodingshan Grottoes is currently underway. The anti‐weathering properties of ambient‐synthesized C‐S‐H‐based mortars will be evaluated through a combination of accelerated aging tests in the laboratory and natural aging tests in relevant application environments. To enable the field application of C‐S‐H‐based materials for the conservation of the Dazu Rock Carvings, additional research on grouting technology and engineering practices will be conducted. This will aim to ensure the effectiveness, compatibility, and long‐term durability of the material in real‐world conservation scenarios.

It should be noted that the current study is focused on laboratory‐scale experiments to evaluate the feasibility and properties of the proposed C‐S‐H‐based material. The images included in this paper are intended to illustrate the potential application of the material in real‐world conservation contexts, rather than to represent actual field tests. Further research, including field trials, is necessary to validate the material's performance under practical conditions.

Despite these limitations, the findings of this study provide valuable insights and guidance for the design, preparation and evaluation of conservation materials for stone heritage, paving the way for future conservation efforts. The synergistic approach of material design, synthesis, property characterization, and interfacial analysis presented here can be broadly applied to screen suitable materials for various applications requiring binding agents, such as cementitious systems and adhesives.

## 4 Experimental Section

### Molecular Dynamics Simulations

The C‐S‐H laminar model was constructed on anhydrous tobermorite 11Å, following the approach proposed by Pellenq et al.^[^ ^38^ ^]^ Random deletion of SiO_2_ monomers from tobermorite ensures proper Si chain polymerization and Q‐distribution, aligning with NMR characterization results. Subsequently, giant canonical Monte Carlo (GCMC) simulations were performed to model the C‐S‐H saturated water uptake state.^[^ ^39^ ^]^ The resulting C‐S‐H model features a layered structure, comprising defective silicon chains, water molecules in the interlayer region, Ca ions and calcium hydroxyl groups. The C‐S‐H model, with a Ca/Si ratio of 1.2 has been validated in prior studies.^[^ ^40^ ^]^ The adsorption model is based on the C‐S‐H superlattice, cut along the interlayer direction, exposing Ca ions, water, and calcium hydroxyls as contact surfaces. Calcite (104) is often employed in studies of stable interfaces,^[^ ^41^ ^]^ and the calcite model illustrated in Figure 1a aligns closely with experimental data. Surface hydroxylated α‐quartz (101)^[^ ^42^ ^]^ and albite (‐1 0 2)^[^ ^43^ ^]^ were faceted along their surfaces and hydroxylated to create stable surface structure models. Quartz and Albite models were then integrated into the C‐S‐H surface to form Quartz/C‐S‐H and Albite/C‐S‐H interfacial composite models, respectively. The C‐S‐H model used in this study has been validated against experimental data in prior work,^[^ ^44^ ^]^ particularly for hydration states and atomic‐level bonding characteristics. Our simulation results—including radial distribution functions (RDFs), coordination numbers, and hydrogen bonding analysis—align with established experimental measurements from X‐ray diffraction (XRD) and nuclear magnetic resonance (NMR) spectroscopy studies of similar systems. The derived RDF profiles and coordination numbers are consistent with experimental observations of bond lengths and interfacial interaction geometries for both C‐S‐H phases and natural sandstone minerals.

The simulations in this study were conducted using LAMMPS^[^ ^45^ ^]^ and employed the ReaxFF force field in an isothermal‐isobaric (NPT) regime. Conventional force fields typically define molecular structures using fixed bond lengths, angles, and torsion angles, which restrict their ability to simulate chemical reactions. In contrast, ReaxFF dynamically characterizes the existence and strength of chemical bonds by calculating bond order (BO'). Bond levels in ReaxFF^[^ ^46^ ^]^ are derived from the instantaneous distances (r_ij_) between atoms (e.g., Equation 1). This allows ReaxFF to adjust bond levels in real‐time, facilitating the formation, breaking, and rearrangement of chemical bonds during simulations.^[^ ^47^ ^]^ Here, r_0_ is the reference distance of the bond (usually the equilibrium bond length), while BO^σ^, BO^π^ and BO^ππ^ represent single, double and triple bonds, respectively.

(1) $$\;\begin{array}{c} {\mathrm{BO}}_{ij}^{\prime }={\mathrm{BO}}_{ij}^{\prime \sigma }+{\mathrm{BO}}_{ij}^{\prime \pi }+{\;\mathrm{BO}}_{ij}^{\prime \pi \pi }=\;\exp\left[p_{\mathrm{bo},1}{\left(\frac{r_{ij}}{r_{0}^{\sigma }}\right)}^{P_{bo,2}}\right]\\ \;\;+\exp\left[p_{\mathrm{bo},3}{\left(\frac{r_{ij}}{r_{0}^{\pi }}\right)}^{p_{bo,4}}\right]+\exp\left[p_{bo,5}{\left(\frac{r_{ij}}{r_{0}^{\pi \pi }}\right)}^{P_{bo,6}}\right] \end{array}$$

This capability enables the simulation to complex molecular dynamics processes and is widely applied to interfacial interactions in intricate systems.^[^ ^48^ ^]^ Currently, the ReaxFF force field is being explored for its structure, mechanical properties, and reactivity in calcium silicate hydrates at the nanoscale.^[^ ^49^ ^]^ The Reaction Force Field (ReaxFF) developed by Van Duin et al.^[^ ^50^ ^]^ is particularly suited for modelling interfacial interactions between C‐S‐H and other materials.^[^ ^51^ ^]^ This system accounts for the interactions between Ca, Si, Al, O, C, and H atoms, which is crucial for accurately modeling bonding in composites. The force field file (ffield.reax.CaSiOH) includes covalent/ionic bonding, bond dissociation, and charge transfer capabilities, which are critical for modeling the dynamic behavior of C‐S‐H, minerals, and interfacial water.^[^ ^52^ ^]^ The ReaxFF force field computes the total potential energy U_sys_ as a summation of multiple energy contributions as shown in Equation 2, including bond energy (U_bond_), van der Waals energy (U_vdWaals_), Coulombic potential energy (U_Coulomb_), under‐coordination energy (U_under_), over‐coordination energy(U_over_), long‐range electron pairs energy (U_lp_), angle energy(U_val_), torsional forces (U_tors_), conjugation effects (U_conj_), and constraint‐based penalty energy(U_pen_).^[^ ^47^ ^]^

(2) $$\begin{array}{ccc} U_{sys} & = & U_{bond}+U_{vdWaals}+U_{Coulomb}+U_{under}+U_{over}+U_{lp}\\ & & +U_{val}+U_{tors}+U_{conj}+U_{pen} \end{array}$$

The molecular dynamics simulations were performed with real units to ensure compatibility with the ReaxFF force field. Periodic boundary conditions were applied in all directions to minimize finite‐size effects and approximate bulk interfacial behavior. The atom style full was employed to explicitly track bonds, angles, and atomic charges, which is essential for bond‐order formalism. Charge equilibration was dynamically resolved via the QEq method, updating atomic charges at every timestep to account for polarization effects. Temperature control was achieved maintaining the system at 300 K to simulate ambient hydration conditions. Short‐range interactions were calculated using a neighbor list with a 2.0 Å cutoff, updated every timestep to ensure accuracy in bond‐order calculations. An equilibrium phase of 3 ns is selected to ensure a stable model of interfacial interactions. Atomic trajectories during this phase are recorded with a data output interval of 0.1 ps, while radial distribution functions (RDF) and coordination numbers (CN) are calculated to characterize the spatial correlation properties of particles at the two interfaces.^[^ ^53^ ^]^

### Materials

Ca(OH)_2_ powders (AR, 95%, Macklin incorporation, Shanghai, China) were used directly without any additional treatment. The SEM images and XRD pattern of Ca(OH)_2_ are presented in Figure S3, Supporting Information, indicating the high purity and layered structure of the Ca(OH)_2_ powders. Three amorphous silica materials were tested in preliminary experiments to identify a suitable active silica. These included two finely dispersed aggregated silica particles with a size of tens of nanometers, one from Baihong Chemical Products Sales Co., Ltd in Zhengzhou, Henan Province (referred to as silica‐1), and another from Macklin incorporation in Shanghai (referred to as silica‐2). Additionally, silica fume with particle sizes ranging from hundreds to thousands of nanometers (98 wt. %, average size of 0.3 µm and specific surface area of 2.0 m^2^ g^−1^) from Henan Platinum Casting Materials Co., Ltd of Henan (referred to as silica‐3) was also tested. Due to its higher reactivity (Figure S3, Supporting Information) and cost‐effectiveness, silica‐1 was selected for this study. Furthermore, quartz sand grains with 250 mesh (99%, Bo Run Casting Materials Co., Ltd, Henan, China) and polycarboxylate superplasticizer (abbreviated as PCE, CQJ‐JSS02, Shanghai Chenqi Chemical Technology Co., Ltd., Shanghai) were used as the filler and superplasticizer, respectively, for the mortars. Sandstone samples were collected from Beishan Grottoes of Dazu Rock Carving in Chongqing, China.

### Synthesis of Calcium‐Silicate‐Hydrate (C‐S‐H) at Room Temperature

The study examined synthesis variables, including C/S ratio (the molar ratio of Ca(OH)_2_ to silica), water/binder ratio, and curing duration. Ca(OH)_2_, silica fume, and deionized water were meticulously blended in proportions, subjected to mechanical stirring at 400 rotations per minute, and subsequently cured within a sealed beaker at ambient conditions (temperature: 22 ± 2 °C, relative humidity: 60 ± 10%).

### Preparation of C‐S‐H Based Mortars

The specific proportions of Ca(OH)_2_, silica‐1, and quartz‐sand grains were gradually blended in a planetary mortar mixer for 120 s. Subsequently, deionized water or water dispersed with PCE was added and mixed slowly for another 120 s, followed by a 30‐s pause. During this interval, mortars adhering to the container wall were scraped into the bottom before all materials were mixed rapidly for another 120 s. To optimize C‐S‐H yield, the thoroughly mixed mortars were poured in 40 × 40 × 160 mm^3^ cuboid molds, sealed with plastic films, and allowed to harden in a chamber at 20 °C and 90 ± 5% R.H. for 28 days.

### Characterization and Testing

The cured samples were collected at intervals of 1 day, 3 days, 7 days, 14 days, and 28 days to analyze the composition and the microstructural features during the pozzolanic reaction. After 28 days, the mechanical strength and pore structures of the cured samples were assessed.

### Characterization and Testing: Fluidity and Setting Time Measurement of C‐S‐H Based Mortars

The fluidity of the fresh mortars was assessed using a cone‐cutting circular mold (height: 60 mm, upper diameter: 50 mm, lower diameter: 35 mm) according to GB/T 2419‐2005,^[^ ^54^ ^]^ without vibration. The mold was placed at the center of a 50 mm × 50 mm glass plate, and the fresh mortar was poured into the mold until it was filled without overflowing. The mold was then lifted, allowing the mortar to flow freely. The flow diameter was measured in four different directions using a ruler, and the final fluidity (mm) was calculated as the average of these measurements. The setting time of the fresh mortars was determined using an Automatic Vicat Apparatus (DL‐AWK, Zhoushan Daolong Technology Co., Ltd) following the procedures specified in GB/T 1346–2011.^[^ ^55^ ^]^ The mortar was poured into a truncated conical mold (top diameter: 65 mm, bottom diameter: 75 mm, height: 40 mm) placed on a glass plate (100 mm × 100 mm). The top of the mold was covered with plastic film to prevent evaporation and maintain moisture. The mold was positioned at the center of the measuring needle of the Vicat apparatus, which was programmed to allow the needle to sink vertically and freely into the mortar at regular intervals. The final setting time (min) was recorded when the penetration depth of the needle was less than 0.5 mm.

### Characterization and Testing: Materials Characterizations

The sandstone samples were initially cut into 25 mm × 25 mm × 5 mm pieces, affixed to glass slides, and subsequently further cut and polished into thin sections measuring ≈0.03–0.04 mm in thickness using a grinding machine (JKPG‐250, Jiangyanqu Jieke Machinery Factory, China). The textural and mineralogical characteristics of the thin section samples were examined using Polarized Light Microscopy with a Leitz Laborlux 12 POL microscope, which was equipped with a Leica DFC280 camera for the acquisition of photomicrographs. Digital Image Analysis (DIA) was conducted on representative micrographs, utilizing Image J software. Grain Size Distribution (hereafter GSD) by determined by estimating minimum Feret (mF), circularity (C = 4π(A/p^2^), where A = area, p = perimeter), and roundness (R = 4×A/(π×maj^2^), where A = area, maj = major axis of inscribed ellipse), which served as shape descriptors of grains.

Mineral compositions of all samples were analyzed using X‐ray Diffraction (XRD) and Thermogravimetric‐Differential Scanning Calorimetry (TG‐DSC). XRD measurements were performed on a Rigaku SmartLab SE X‐ray diffractometer, equipped with a HyPix‐400 2D detector, with the following measurement parameters: Cu‐Kα radiation, wavelength λ = 1.5405Å, accelerating voltage 40 kV, current 15 mA and 5–70° 2θ exploration range with a step size of 0.02° 2θ. Mineral phases were identified by using the Joint Committee on Powder Diffraction Standards (JCPDS).

TG‐DSC analyses were conducted simultaneously using a Mettler Toledo TGA/DSC 3+ instrument, equipped with STARe software. ≈20 mg samples were placed in closed 70 µL Pt crucibles and heated from 25 to 1000 °C at a rate of 10 °C min^−1^ under flowing N_2_ at a rate of 50 mL·min^−1^.

Microstructural features of the C‐S‐H based mortars and the interface of C‐S‐H/sandstone were determined through two methods: (i) Transmission Electron Microscopy (TEM) was conducted using a JEM‐F200 flash instrument (JEOL), operating at a 200 kV acceleration voltage. For C‐S‐H‐based mortars, the powdered samples were dispersed in ethanol and sonicated for 30 s before being collected on the carbon‐coated Cu grids (Electron Microscopy China). Selected Area Electron Diffraction (SAED) and energy‐dispersive X‐ray spectrometer (EDS) patterns were performed to identify the C‐S‐H phase. High‐resolution (HR) TEM SAED were also performed on the C‐S‐H/Sandstone interface samples. The C‐S‐H mortar‐bonded sandstone sample was meticulously sectioned and embedded in epoxy resin to acquire high‐quality HR‐TEM images. A 1 mm‐thick slice was then extracted from the resin‐embedded sample and polished down to ≈40 µm. Ion thinning was performed using a Gatan 685 instrument at an angle of 10 degrees and an acceleration voltage of 4 kV for 5 h, after which the thin section was attached to a copper ring. Further thinned produced ultrathin sections ranging from tens to hundreds of nanometers in thickness, achieved by applying an angle of 5 degrees and an acceleration voltage of 2.5 kV for 30 min. (ii) Scanning Electron Microscopy (SEM) images of fresh‐fractured specimens were acquired using a Zeiss Gemini SEM300 field emission microscope, which was equipped with an Oxford Instruments model X‐Max^N^ 51‐XMX1140 energy‐dispersive X‐ray spectrometer (EDX). Gold sputtering was applied using a Quorum SC7620 to enhance electrical conductivity. Spectra and maps of characteristic X‐ray photon emission energies of elements were obtained using the EDX system.

### Characterization and Testing: Mechanical and Physical Properties of the Cured Mortars

The flexural strength and compressive strength of the prism bulks of C‐S‐H mortars and sandstone samples were determined using an Electromechanical Universal Testing Machine. An MTS Electromechanical Universal Testing Machine (E44.304) was used for the three‐point flexural‐tensile strength test. Specimens were positioned on supports with a distance of L (mm) and subjected to loading at a rate of 1.27 mm min^−1^ until they fractured at a maximum force F_t_ (in N). Flexural strength (in megapascals, MPa) was calculated by 1.5 × F_t_ × L divided by the cubic length of the fracture cross‐section, denoted as b^3^ (mm^3^). The compressive strength test was carried out on two fragments of each beam specimen that fractured during the flexural‐tensile strength test, employing the same machine and load rate. Compressive strength (MPa) was calculated as the maximum load F_c_ (N) divided by the cross‐section area, denoted as b^2^ (mm^2^). Three specimens were tested in parallel for flexural strength, while six specimens were tested for compressive strength.

Porosity and pore size distribution data were obtained through Mercury Intrusion Porosity measurement (MIP). For MIP analysis, lime mortar samples were first dried at 105 °C for 24 h, then cut into small fragments, and subsequently tested using an Autopore V9620 instrument (Micromeritics Instrument Corporation).

To evaluate the adhesive strength of C‐S‐H based mortars with natural stones, sandstones from Beishan Grottoes of Dazu Rock Carvings were cut into 40 × 40 × 10 mm^3^ cuboids for pullout adhesive strength tests (Figure S6a, Supporting Information) and two different sections for shear bond strength tests (Figure S6b, Supporting Information). Pullout adhesive strength (MPa) was performed by a HC‐D10 Pull‐off Adhesion Tester (HICHANCE) determined by dividing the maximum force (F_m_ / N) by the adhered area (40 × 40 mm^2^), while shear bond strength (MPa) was performed by MTS‐E44.304 and calculated by dividing the breaking load (F_s_ / N) by the product of length (63 mm) and width (38.5 mm) of the grouted area, following the evaluation method outlined by the Getty Conservation Institute.^[^ ^56^ ^]^

The coefficient of permeability of the hardened C‐S‐H mortars was evaluated in accordance with the standard method of MT224‐1990.^[^ ^57^ ^]^ Three cylindrical specimens, each with a diameter and height of 50 mm, were prepared and cured at 20 °C and 90 ± 5% R.H. for 28 days prior to test. The permeability measurements were conducted using a HYS‐4S rock permeability analyzer. The reported coefficient of permeability for the C‐S‐H based mortars is the arithmetic mean of three tested samples.

## Conflict of Interest

The authors declare no conflict of interest.

## Supporting information



Supporting Information

Supplemental Movie 1

## Data Availability

The data that support the findings of this study are available in the supplementary material of this article.
