# Supplementary material for: From Ancient Techniques to Modern Solutions: In Situ Synthesis of C‐S‐H for Sandstone Conservation
Source: Adv Sci (Weinh). 2025 May 9;12(29):2503333. doi: 10.1002/advs.202503333 (PMC12362824; doi:10.1002/advs.202503333)
Supplement: Supplementary file 1 — Supporting Information [file ADVS-12-2503333-s001.pdf]

ADVANCED SCIENCE
Open Access

Supporting Information

for *Adv. Sci.*, DOI 10.1002/advs.202503333

From Ancient Techniques to Modern Solutions: In Situ Synthesis of C-S-H for Sandstone Conservation

Mengjun Jia, Huimin Yan, Qingqing Xu, Celestino Grifa, Gang Zhao, Siwei Jiang, Jinhua Wang, Zhenhua Wei*, Han Liu* and Xiao Ma**

Supporting Information

From Ancient Techniques to Modern Solutions: In-Situ Synthesis of C-S-H for Sandstone Conservation

Mengjun Jia, Huimin Yan, Qingqing Xu, Celestino Grifa, Gang Zhao, Siwei Jiang, Jinhua Wang, Zhenhua Wei*, Han Liu*, Xiao Ma**

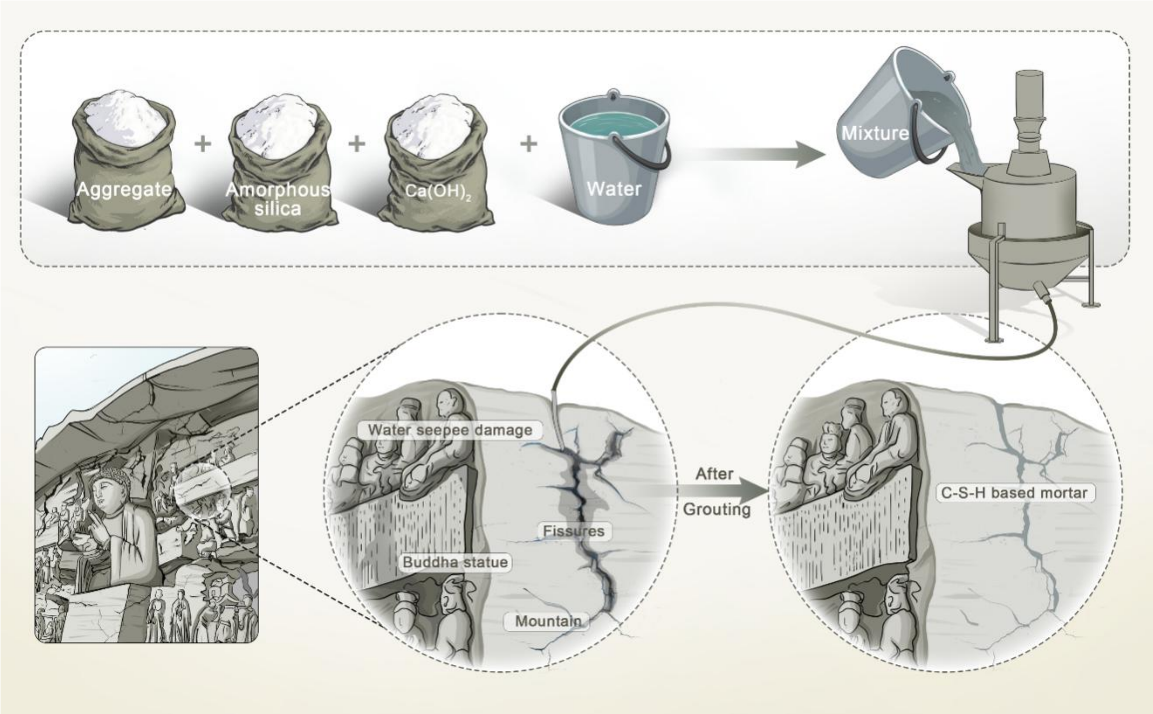

Figure S1. The schematic drawing for the preparation and on-site application of Calcium-Silicate-Hydrate (C-S-H) based grouting mortars on Dazu Rock Carvings

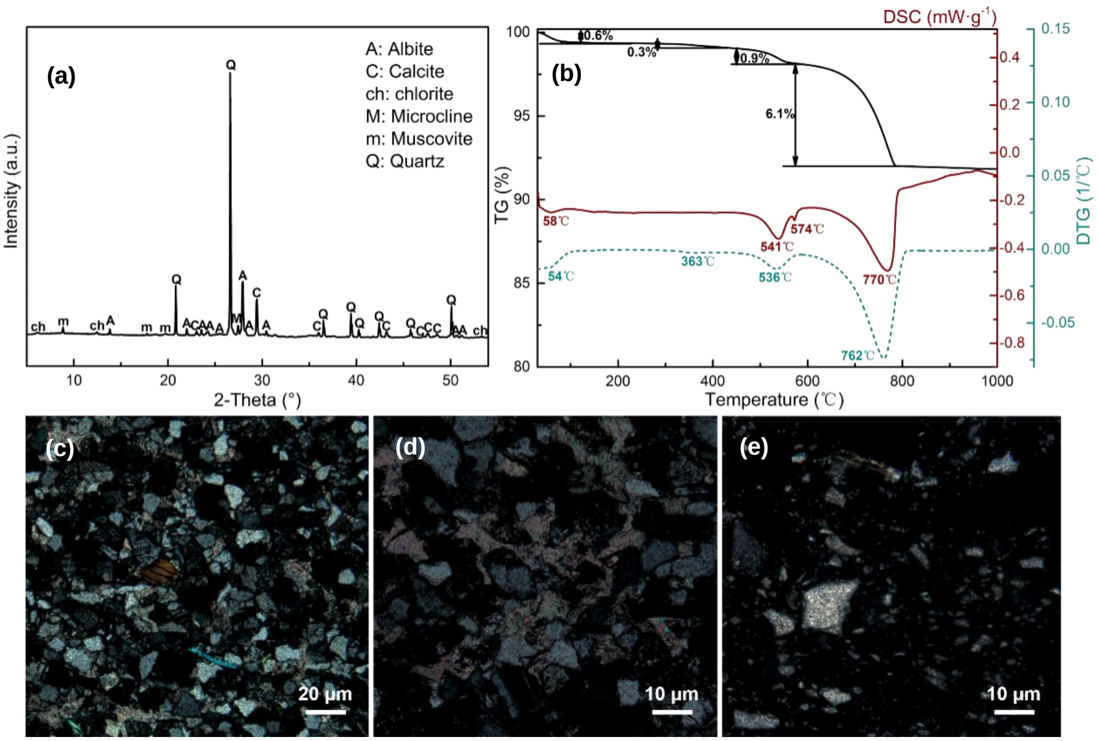

Figure S2. The analysis of sandstones collected from the Beishan Grottoes of Dazu Rock Carving includes: (a) XRD analysis; (b) TGA-DSC-DTG analysis; (c) Thin section micrographs depicting quartz, feldspar, and muscovite as main minerals in Beishan sandstone under crossed polars; (d) Thin section micrographs showing calcite binding medium among the quartz and feldspar grains in Beishan sandstone under crossed polars; (e) Thin sections micrographs displaying quartz grains scattered in the isotropic matrix of C-S-H based mortars S14 under crossed polars. Strictly speaking, from a petrological classification perspective, the Beishan stone should be categorized as "siltstone." However, for the sake of simplicity and common understanding, the term "sandstone" is used throughout the manuscript.

Dazu Rock Carvings (大足石刻 in Chinese), is a UNESCO World Heritage Site located in Dazu District, Chongqing, China. Constructed from the late 9th century to the mid-13th century CE, these carvings include more than 100,000 statues that vividly reflect the social life of the time, from secular to religious themes. These carvings are considered representative works of Late Taoist cave art in China. Among them, the statue groups carved at the five major sites—Beishan (北山 in Chinese), Baodingshan (宝顶山 in

Chinese), Nanshan (南山 in Chinese), Shizhuanshan (石篆山 in Chinese), and Shimenshan (石门山 in Chinese)— are the most renowned.

Beishan grottoes are carved out of the greyish or reddish fine-grained stone, geologically dating back to the Jurassic period and formed in a fluvial and lacustrine shallow-water depositional environment.[58] The characteristic feature of these stones are gentle bedding planes with interbedded layers of sandstone and mudstone. The later-formed, gently-dipping anticline geological structure resulted in the development of two sets of tensional fractures within the rock mass oriented NNE 10° to 20° and NWW 280° to 300°.These fractures range from 80 centimeters to 1 centimeter in width, creating preferential pathways for water infiltration.[59]

The GSD for the sandstone collected from Beishan grottoes, evaluated by Feret and minimum Feret diameters (Table S3), varies within the intervals of 4.1-27.2 microns (average 10 microns) and 1.9-18.2 microns (average 5.6 microns), respectively. These values fall within the ranges of very-fine silt to medium silt on the Wentworth scale.[60] The average roundness (R) and circularity (C) both measure at 0.6. Based on: i) the relative proportion of rock-forming minerals (quartz and feldspar) and lithic fragments, ii) the absence of matrix, and iii) the GSD, the rock can be classified as quartz-sandstone. On the other hand, subangular quartz crystals (R = 0.5, C = 0.6) predominate in the C-S-H-based mortar material (**S14**), with sporadic prismatic crystals of feldspar (orthoclase) (see Figure. S4E). The GSD is slightly finer compared to the rock, ranging from 0.4-12.3 microns (fine silt) and 0.3-5.2 microns (very-fine silt) for Feret and minimum Feret diameters, respectively (refer to Table S3). The texture is clearly bimodal, with crystals scattered in the isotropic matrix of C-S-H, which includes sub-micrometric birefringent particles. Rounded macropores up to 150 microns in size and elongated fractures are present.

From a mineralogical standpoint, the sandstones found in the Beishan Grottoes of Dazu Rock Carvings primarily consist of quartz, feldspar, and calcite (~14 wt. %), with minor occurrences of illite/muscovite and chlorite, as revealed by the integration of XRD and TGA-DSC-DTG data (**Figure S2a,b**). This comprehensive mineralogy was further confirmed through examination under a polarized light microscope, which indicated that

quartz is the most abundant mineral, appearing as sub-rounded monocrystalline crystals. Following quartz, feldspar is present, displaying prismatic forms with orthoclase, microcline, and lower plagioclase optical characteristics. Additionally, minor deformed chlorite and muscovite lamellae and sporadic lithic fragments (such as chert) were observed (**Figure S2c**). The rock's structure is grain-supported, featuring concave/convex contacts among the grains, indicating thorough compaction, with most of the pores filled by interstitial calcite (**Figure S2d**). The collected sandstones' compressive and flexural strength are measured to be 68.6 ± 7.3 MPa and 5.0 ± 0.3 MPa, respectively (**Table S2**).

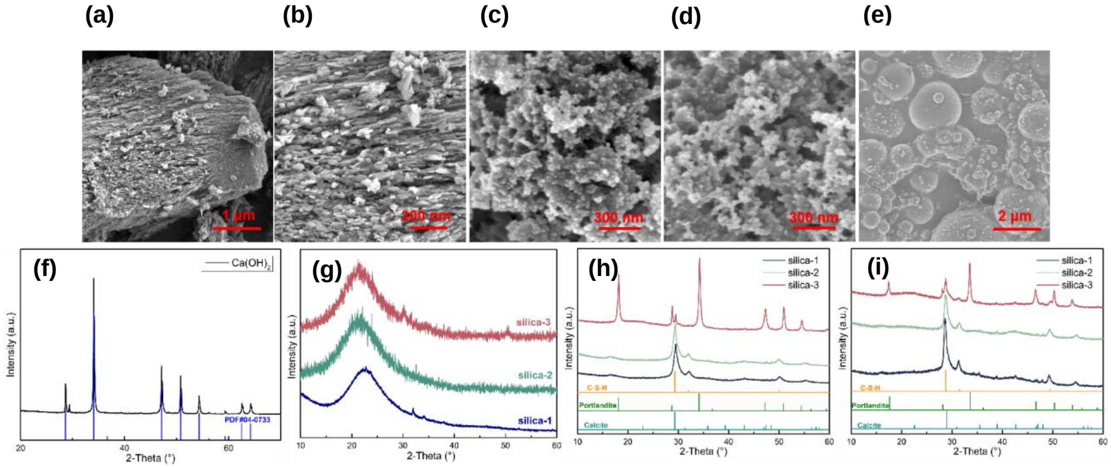

Figure S3. SEM images for the raw materials of $Ca(OH)_2$. (a-b) and amorphous silica (c) silica-1, (d) silica-2, (e) silica-3, respectively. (f) XRD pattern of $Ca(OH)_2$ powders. (g) shows the XRD pattern of the tested silica, indicates their amorphous characteristics.(h) and (i) depict the XRD pattern of the products in $Ca(OH)_2$-silica mixed mortars with a Ca/Si molar ratio of 1 and a water/solid ratio of 6 after curing for 7 days and 28 days, respectively. These patterns demonstrate the higher reactivity of smaller-sized silica-1 and silica-2 compared to silica-3. Considering commercial prices, silica-1 was selected for use in this research.

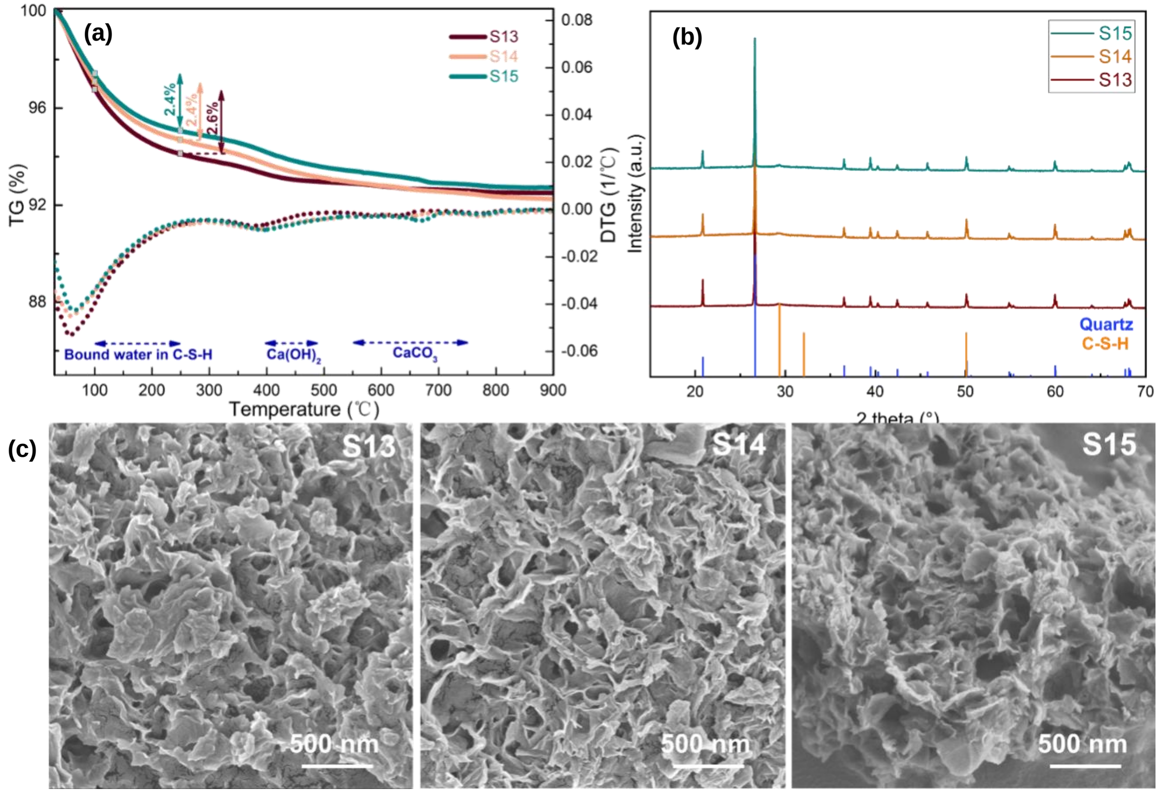

Figure S4. (a) TG-DTG analysis, (b)XRD analysis, and (c) microstructure of C-S-H-based mortars S13-S15 with a C/S ratio of 0.8, water/binder ratio of 2, binder/aggregate ratio of 1:3 and PCE content of 3-5 wt. % after curing for 28 days.

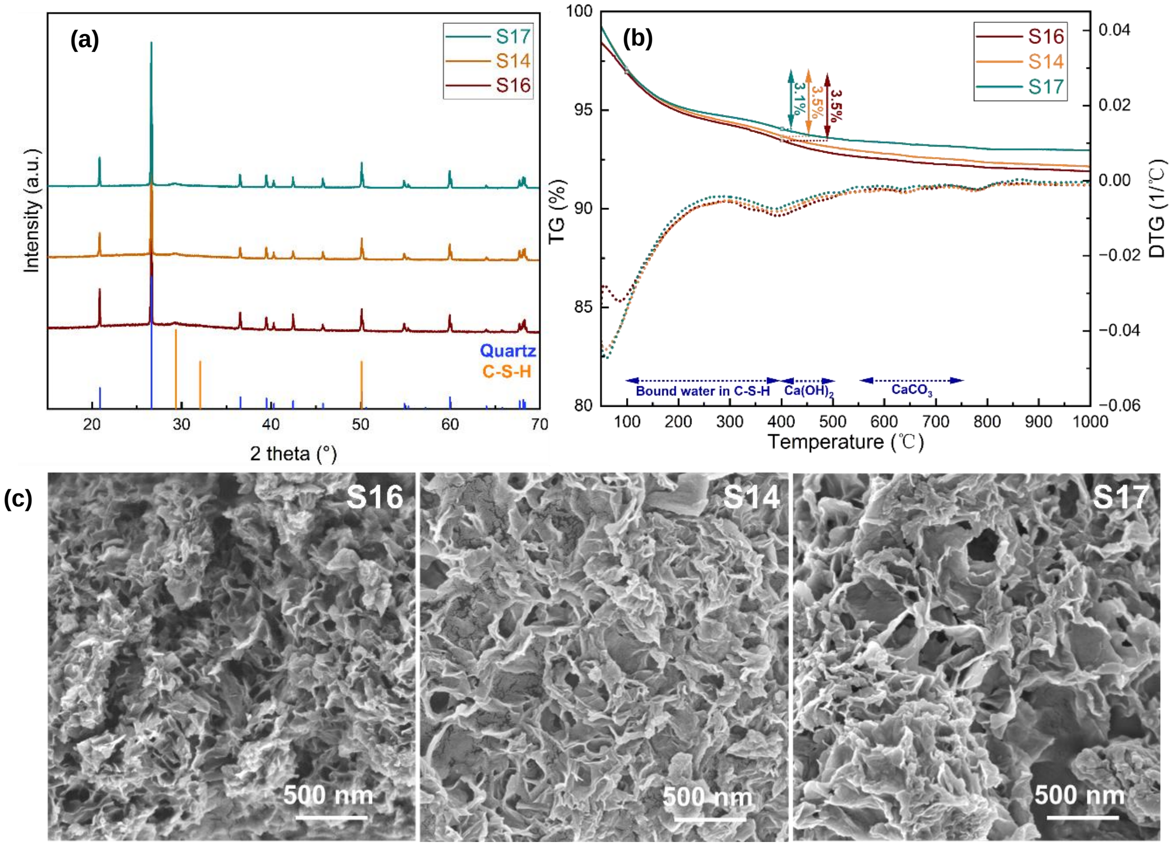

Figure S5. (a)XRD anlysis, (b) TG-DTG analysis, and (c) microstructure of C-S-H based mortars S16, S14 and S17 with a C/S ratio of 0.8, water/binder ratio of 1.5-2.5, binder/aggregate ratio of 1:3 and amount of PCE of 4 wt. % after curing for 28 days.

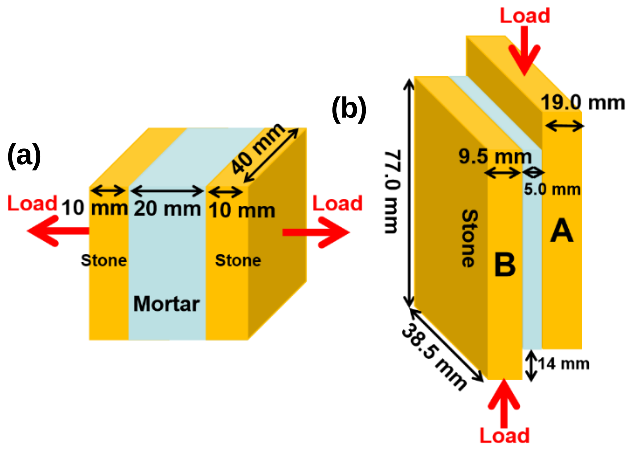

Figure S6. Schematic drawing of the samples configurations for the pullout adhesive strength test (a) and shear bond strength test (b) [56]

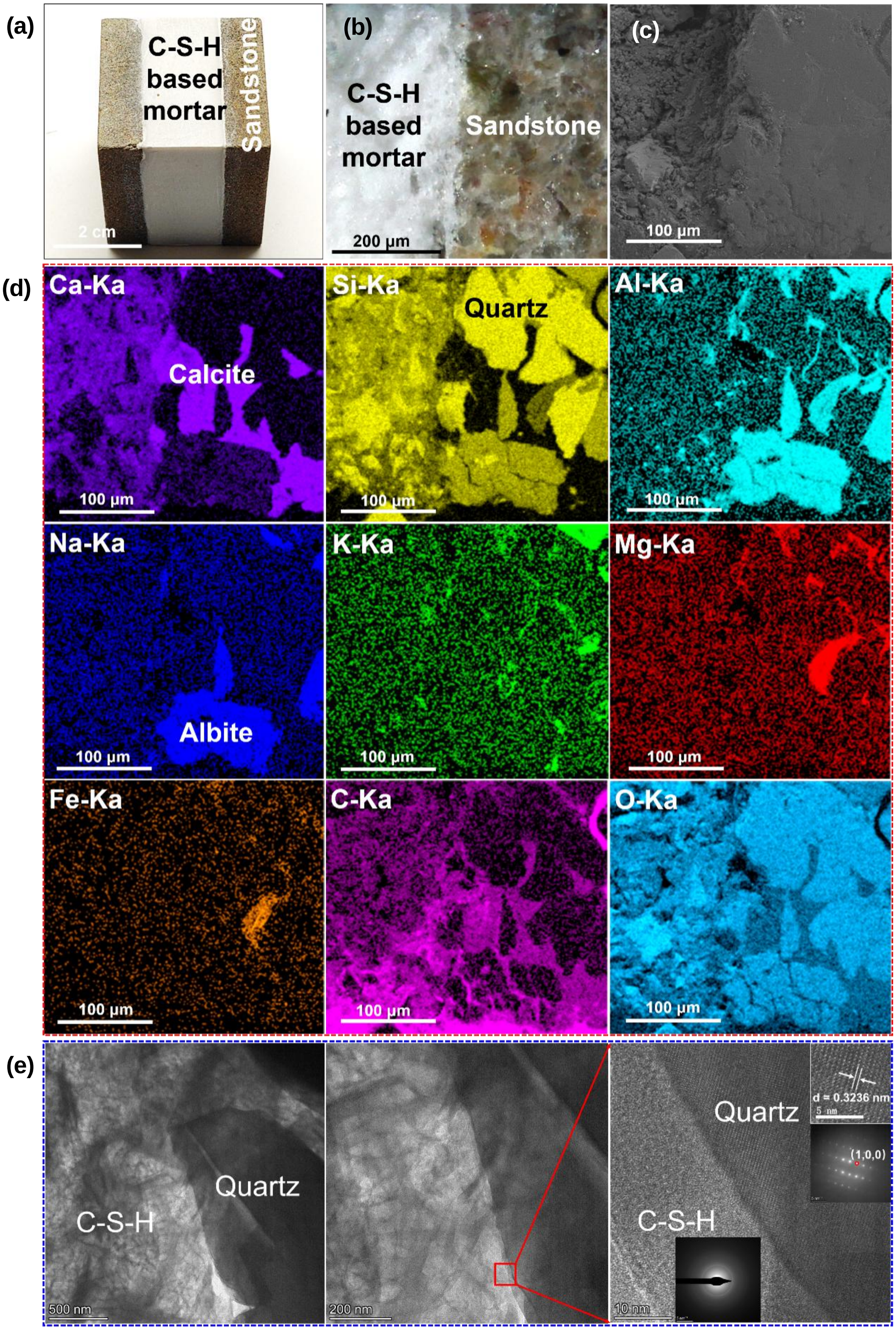

Figure S7. Macro and micro images of the C-S-H/Sandstone bonding samples (a) Macro-

appearance for the sandwich binding samples of C-S-H based mortar and sandstone; (b) Optical image of the interface between C-S-H based mortar and sandstone; (c) Secondary Electron (SE) image of the same interface; (d) Element mapping of the C-S-H/Sandstone interface; (e) High-resolution TEM images of the interface between the amorphous C-S-H phase and crystalline α-quartz phase.

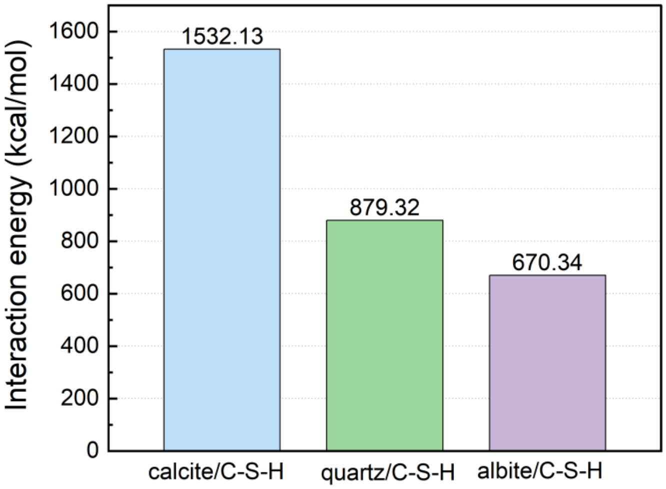

Figure S8. Interaction energy of calcite/C-S-H, quartz/C-S-H and albite/C-S-H

Figure S8 compares the relative interaction strengths between C-S-H and each mineral phase. The calculated interaction energies are 1532.13 kcal/mol for the calcite/C-S-H interface, 879.32 kcal/mol for quartz/C-S-H, and 670.34 kcal/mol for albite/C-S-H. These results indicate that calcite forms the strongest bond with C-S-H, followed by albite and quartz. This finding has important implications for material interfacial behavior, particularly in stone conservation, where the C-S-H/mineral interface governs grout adhesion and stability on stone surfaces.

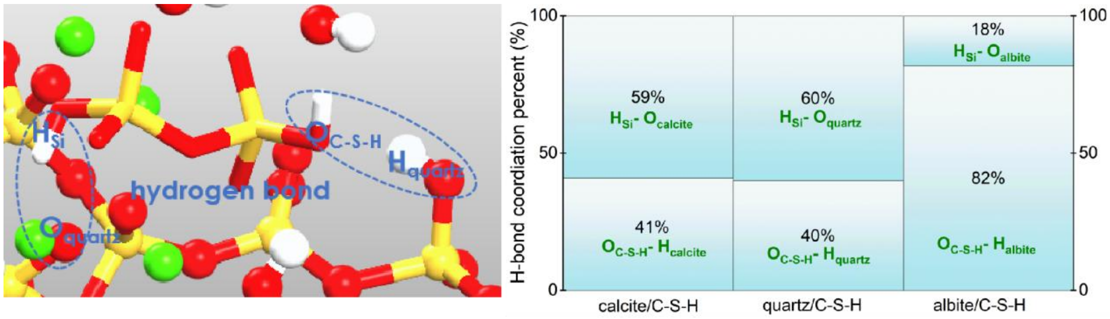

Figure S9. Statistical composition of interfacial hydrogen bond

As shown in **Figure S9**, the proportions of these two hydrogen bond types were calculated for each interface. The results reveal that the calcite/C-S-H interface is dominated by H_{Si}-$O_{calcite}$ bonds, indicating that C-S-H primarily donates hydrogen to oxygen atoms on the calcite surface. Similarly, at the quartz/C-S-H interface, C-S-H preferentially donates hydrogen to the quartz surface, resulting in a higher number of hydrogen bonds. In contrast, at the albite/C-S-H interface, hydrogen bonding is primarily mediated by $O_{C\text{-}S\text{-}H}$-H_{albite} interactions, suggesting that albite acts as the hydrogen donor while C-S-H accepts hydrogen. These findings highlight the distinct hydrogen bonding behaviors at the different interfaces, providing a comprehensive understanding of the structural and chemical interactions in these systems.

Table S1. TGA analysis of C-S-H based mortars

No.	Initial C/S	W/B ratio	B/A ratio	TGA (wt. %)		
				Dehydration of C-S-H	Mass of $Ca(OH)_2$	Mass of $CaCO_3$
				105-400 ℃	400-480 ℃	550-750℃
**S8**	0.8	2	1:3	3.0	0.0	0.9
**S9**	1.0	2		2.9	0.0	1.1
**S10**	1.2	2		2.8	0.0	2.3
**S11**	1.5	2		2.6	0.0	2.5
**S12**	2.0	2		2.6	2.9	3.2

Note: The data presented in this table is derived from **Figure 3b**.

Table S2. The mechanical strength and porosity of the sandstone collected from the Beishan Grottoes of Dazu Rock Carvings

Samples	Compressive Strength (MPa)	Flexural Strength (MPa)	Ultrasonic Velocity ($m \cdot s^{-1}$)		Porosity (%)
Sandstone	68.6 ± 7.3	5.0 ± 0.3	A	2601 ± 74	9.7
			B	1673 ± 108	
			C	1626 ± 80	

*Considering the anisotropy of sandstones, A/B/C are used to mark three different directions. Among which, A is parallel to the lineation of sandstone and B/C are two orthogonal directions to lineation.

Table S3. The grain size distribution (GSD) of the sandstone collected from the Beishan Grottoes of Dazu Rock Carvings and the C-S-H based **S14** samples

Sandstone	Feret	MinFeret	C	R
min	4.13	1.94	0.12	0.13
max	27.20	18.23	0.91	0.98
average	9.98	5.62	0.63	0.57
st.dev	3.78	2.28	0.12	0.17

C-S-H based mortar	Feret	MinFeret	C	R
min	0.41	0.29	0.36	0.23
max	12.39	5.26	0.99	0.95
average	2.59	1.30	0.66	0.54
st.dev	2.11	0.94	0.13	0.16

Movie S1. The scheme for grouting the C-S-H-based mortars into the fissures of Dazu Rock Carvings
